# Sensing and adhesion are adaptive functions in the plant pathogenic xanthomonads

**DOI:** 10.1186/1471-2148-11-67

**Published:** 2011-03-11

**Authors:** Nadia Mhedbi-Hajri, Armelle Darrasse, Sandrine Pigné, Karine Durand, Stéphanie Fouteau, Valérie Barbe, Charles Manceau, Christophe Lemaire, Marie-Agnès Jacques

**Affiliations:** 1 UMR077 PaVé, INRA, 42, rue Georges Morel, F-49071 Beaucouzé, France; 2 CEA/DSV/IG/Genoscope, 2 rue Gaston Cremieux, 91057 Evry Cedex 06, France; 3 UMR077 PaVé, Université d'Angers, 42, rue Georges Morel, F-49071 Beaucouzé, France

## Abstract

**Background:**

Bacterial plant pathogens belonging to the *Xanthomonas *genus are tightly adapted to their host plants and are not known to colonise other environments. The host range of each strain is usually restricted to a few host plant species. Bacterial strains responsible for the same type of symptoms on the same host range cluster in a pathovar. The phyllosphere is a highly stressful environment, but it provides a selective habitat and a source of substrates for these bacteria. Xanthomonads colonise host phylloplane before entering leaf tissues and engaging in an invasive pathogenic phase. Hence, these bacteria are likely to have evolved strategies to adapt to life in this environment. We hypothesised that determinants responsible for bacterial host adaptation are expressed starting from the establishment of chemotactic attraction and adhesion on host tissue.

**Results:**

We established the distribution of 70 genes coding sensors and adhesins in a large collection of xanthomonad strains. These 173 strains belong to different pathovars of *Xanthomonas *spp and display different host ranges. Candidate genes are involved in chemotactic attraction (25 genes), chemical environment sensing (35 genes), and adhesion (10 genes). Our study revealed that candidate gene repertoires comprised core and variable gene suites that likely have distinct roles in host adaptation. Most pathovars were characterized by unique repertoires of candidate genes, highlighting a correspondence between pathovar clustering and repertoires of sensors and adhesins. To further challenge our hypothesis, we tested for molecular signatures of selection on candidate genes extracted from sequenced genomes of strains belonging to different pathovars. We found strong evidence of adaptive divergence acting on most candidate genes.

**Conclusions:**

These data provide insight into the potential role played by sensors and adhesins in the adaptation of xanthomonads to their host plants. The correspondence between repertoires of sensor and adhesin genes and pathovars and the rapid evolution of sensors and adhesins shows that, for plant pathogenic xanthomonads, events leading to host specificity may occur as early as chemotactic attraction by host and adhesion to tissues.

## Background

Deciphering how bacteria adapt to their hosts helps explain how they spread. Host specificity can be established by determining the genes coding virulence factors that are not conserved among strains, which differ in their host range [1]. Virulence-associated genes are expressed during initial host colonisation, multiplication, development of symptoms, and dispersal. Sarkar and colleagues [1] and Hajri and associates [2] demonstrated that canonical virulence factors such as type III effectors (T3Es) play a critical role in host specificity. T3Es, however, are injected into plant host cells once bacteria have already penetrated into host tissues [3]. Thus, phases preceding infection could also be involved in host specificity and therefore be under selective pressures.

For bacteria to adapt specifically to their hosts, they sense favourable environmental stimuli and then they move toward favourable conditions [4,5]. Bacteria have evolved receptors and sensors in their cell walls to detect chemical and environmental signals such as the presence of chemoattractants, chemorepellents, and oxygen. They thereby integrate information on their biotic and abiotic environment [6]. Studies on *Rhizobia *revealed the importance of sensors in the perception of specific host signals early during symbiotic interaction with legumes [7,8]. Similarly, *Agrobacterium tumefaciens *and *Ralstonia solanacearum *specifically detect various components from root exudates that attract them toward their hosts [9,10].

Environmental signals are mainly detected by Methyl-accepting Chemotaxis Proteins (MCPs) and Sensors of Two-Component Regulatory System (STCRS). MCPs are the principal components of the chemotaxis system [4]. Detection of signals by these transmembrane chemoreceptors directs cell locomotion by regulating the histidine kinase CheA, which in turn communicates the information to the flagellar motor by phosphorylating its cognate response regulator CheY [4,5]. Changes in the direction or the speed of flagellar rotation modify swimming behaviour, resulting in movement towards higher gradients of attractants and away from high concentrations of repellents [11,12]. In *Escherichia coli*, chemotaxis proteins cluster in membrane-associated patches [13,14]. Interactions within patches contribute to the notable features of this signalling system: high sensitivity, wide dynamic range, signal integration, memory, and adaptation [15]. Besides MCPs, bacteria sense their nutritional environment through TonB-Dependent Transporters (TBDTs) [16]. A large proportion of TBDT genes are related to plant scavenging and carbohydrate utilisation. TBDTs are over-represented in various bacteria interacting with plants such as *Xanthomonas *spp. [17].

Adhesion to a surface is a prerequisite for aggregation in a biofilm, which enhances the resistance of bacteria to various biotic and abiotic stresses, favours the coordination of adapted responses to environmental changes, and allows multiplication [18,19]. Sensing and adhesion mechanisms are interconnected since biofilm formation is regulated by a chemosensory system [20]. The adhesion step involves surface structures in a broad group of fimbrial and nonfimbrial adhesins. The fimbrial proteins include type IV pili (Tfp), which are polymeric assemblies of the protein pilin [21,22]. The nonfimbrial adhesins belong to the autotransporter family (*e.g*. XadA and YadA proteins) [23,24] and to the two-partner secretion system (*e.g*. FhaB and YapH proteins) [25].

Each plant pathogenic bacterium belonging to the *Xanthomonas *genus is able to colonise a restricted variety of plant hosts and microniches. *Xanthomonas *are exclusively plant-associated bacteria, mainly phyllosphere colonisers, and are not encountered in other environments [26]. Globally, they infect a huge range of economically important plants such as rice, banana, citrus, bean, tomato, pepper, sugarcane, and wheat [26]. The large host range of the genus strikingly contrasts with the typically narrow host range of individual strains restricted to one or several species of a botanical family [27]. Besides their very homogeneous phenotype, xanthomonads differ mainly by their host specificity. This is illustrated in the pathovar subspecific division, which clusters bacterial strains causing similar symptoms on a same host range [28]. A few pathovars are represented by polyphyletic genetic lineages *i.e. *pv. *phaseoli *[29] and pv. *dieffenbachiae *[30]. The two lineages of the latter pathovar are pathogenic on different hosts (*Anturium *and *Dieffenbachia*) and hence may be considered as separate pathovars [30].

The 12 available *Xanthomonas *genomes http://www.genomesonline.org/ revealed a huge number of genes encoding chemotactic sensors, systems sensing the nutritional environment of the cell (MCPs, TBDTs and STCRS), and attachment structures [17,31,32]. This large number reflects a high degree of adaptability and the presence of mechanisms and structures involved in the exploration of the bacterial environment and adaptive colonisation. This led us to hypothesize that determinants responsible for bacterial host specificity are expressed starting from the establishment of chemotactic attraction by host tissues and adhesion on phylloplane. Thus, characterizing repertoires of genes encoding sensors and adhesins will provide information about the interaction and the adaptation of bacteria to their host plants. Here we characterized the distribution of genes encoding MCPs, STCRS, TBDTs, and adhesins in a large collection of strains belonging to different pathovars in several species of *Xanthomonas*. We also tested for molecular signatures of selective pressures on candidate genes. Two types of outcomes were expected: (i) strong purifying selection acting on genes involved in recognition of common structures of plant tissues and (ii) adaptive divergence on genes coding for sensors and adhesins used for colonisation of specific niches. We identified a large variety of repertoires generally fitting with the pathovar clustering. Adaptive divergence was found to affect most candidate genes. These findings provide insight on the evolutionary importance of chemotactic attraction and adhesion in the host specificity of plant pathogenic bacteria.

## Results

### Identification and selection of genes

Based on data mining, we identified genes involved in bacterial attraction, sensing, and adhesion to host. We extracted sequences of genes from four complete genome sequences (*X. axonopodis *pv. *vesicatoria *(*Xav*) strain 85-10, *X. axonopodis *pv. *citri *(*Xac*) strain 306, *X. campestris *pv. *campestris *(*Xcc*) strain ATCC33913, and *X. oryzae *pv. *oryzae *(*Xoo*) strain KACC10331). These four bacteria have different host ranges and are phylogenetically distant. First, a list containing 320 genes involved in sensing, chemotaxis, motility, and adhesion was established using BLAST analysis. This list includes 30 genes encoding MCPs, 17 genes encoding chemotactic protein, 34 genes encoding flagellar components, 31 genes involved in Tfp biogenesis, 10 genes encoding nonfimbrial adhesins, 115 genes encoding TBDTs, and 83 genes encoding STCRS (See additional file 1: Table A1 for the complete list of genes involved in sensing and adhesion in xanthomonads). Second, for selection detection analyses, we selected genes that were ubiquitous in the four genomes, for which polymorphism was observed and that encodes proteins acting upstream in regulation cascades. Hence, we selected 70 candidate genes: 28 genes encoding TBDTs, 7 genes encoding STCRS, 25 genes encoding MCPs, 2 genes encoding Tfp sensors, 1 gene encoding Tfp assembly ATPase, and 7 genes encoding adhesins.

### Characterization of repertoires of MCPs, STCRS, TBDTs, and adhesins

We investigated the distribution of the 70 selected genes in 173 strains belonging to different lineages in *Xanthomonas *spp. by PCR (See additional file 2: Table A2 for the list of bacterial strains). Three independent PCR reactions with two different sets of primers and three DNA batches were used to monitor the presence of genes in the strain collection. A gene was considered absent when no signal was obtained. But with this approach, one cannot rule out that some genes that we considered as potentially absent may have undergone several point mutations in the primer regions, which could result in divergence sufficient to prevent amplification through several PCRs. Every PCR result on the DNA of *X. axonopodis *pv. *phaseoli *GL *fuscans *strain CFBP4834 was confirmed by BLAST analysis on the draft genome sequence of this strain (our unpublished data). No discrepancies were observed for this strain in any of the 70 candidate genes.

**Figure 1:**
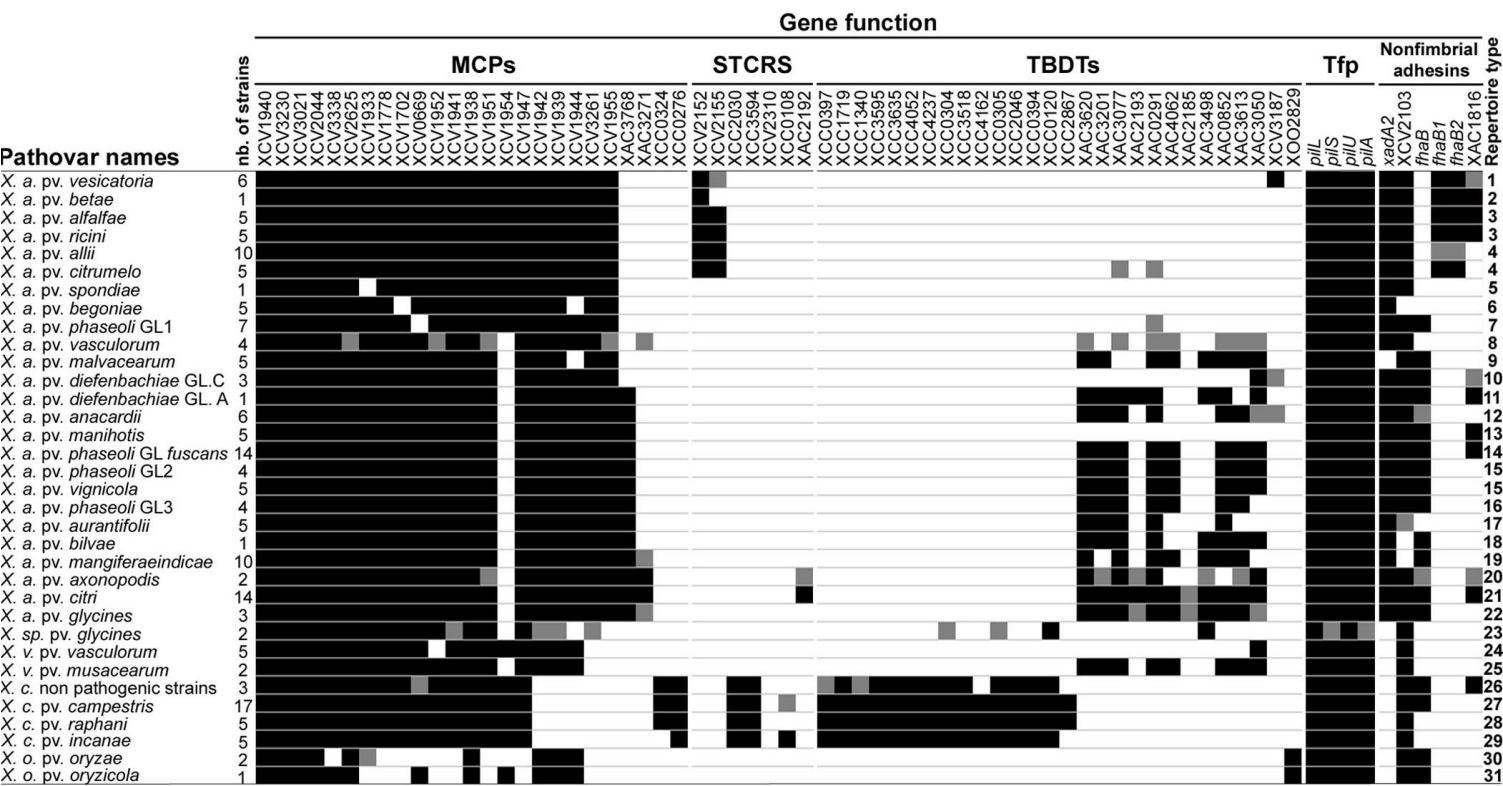
**Distribution of genes involved in sensing and adhesion among lineages of *Xanthomonas *spp**. Black and white squares represent the presence and the absence, respectively, of the corresponding gene in at least 80% of strains of a pathovar or a genetic lineage. Grey squares indicate that the presence of the gene is variable among strains of the lineage. Thirty one repertoire types were identified on the basis of the presence-absence of genes under study. At least three independent PCR reactions were performed to ascertain the presence/absence of each gene. *X. a*.: *X. axonopodis*; *X. v*.: *X. vasicola*; *X. c*.: *X. campestris *and *X. o*.: *X. oryzae*.

MCPs, STCRS, TBDTs, and adhesin repertoires contained three categories of genes based on their presence/absence (Figure 1): first, ubiquitous genes showing a broad distribution among strains (*e.g*. XCV1940, detected in all strains, and *pilS*, detected in most strains); second, genes displaying a variable distribution since they were not detected in several strains (*e.g*. XCV1954 and XCV2103); third, genes found in only one species (*e.g*. XAC3768 and *xadA2*, detected only in *X. axonopodis*, and XCC0324 and XCC0276, found only in *X. campestris*).

Most bacterial lineages in *Xanthomonas *spp. displayed unique repertoires of MCPs, STCRS, TBDTs, and adhesins (Figure 1). Our results showed that 28 of the 34 pathovars and genetic lineages of *Xanthomonas *spp. had distinct repertoires. In contrast, different pathovars may display the same repertoire, as do pathovars *alfalfae *and *ricini*, pathovars *allii *and *citrumelo*, and pathovars *vignicola *and genetic lineage GL2 of pathovar *phaseoli*. A large variability in the number of genes constituting repertoires was observed among pathovars with *X. oryzae *strains harbouring the smallest repertoires, whereas strains of *X. axonopodis *pv. *citri *displayed the largest repertoires.

Repertoires of MCPs, STCRS, TBDTs, and adhesins were almost identical within pathovars *alfalfae*, *begoniae*, *malvacearum*, *manihotis*, *musacearum*, and *oryzae*. Regarding pathovar *anacardii*, strains isolated from *Mangifera indica *(CFBP2913 and CFBP2914) displayed repertoires that differed by three genes (XAC3050, XCV3187 and *fhaB*) from the repertoires of the strains isolated from *Anacardium occidentale *(CFBP7240, CFBP7241, CFBP7242 and CFBP7243). The diversity observed among repertoires in pathovar *allii *could not be associated with either the genetic diversity or the known host of isolation [33] (See additional file 2: Table A2 for the list of host of isolation of each bacterial strain).

**Figure 2:**
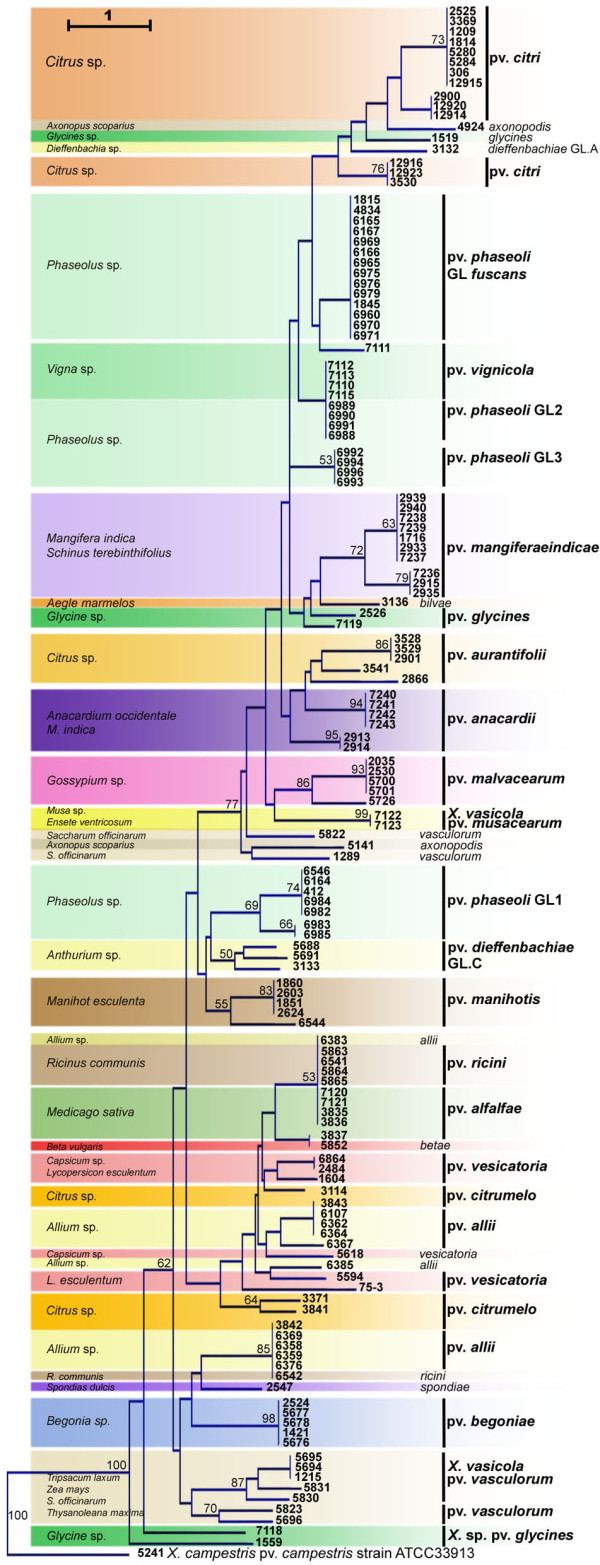
**Dendrogram constructed based on presence/absence of candidate genes in strains of *Xanthomonas *spp**. The dendrogram was constructed with the Neighbour-Joining method using Euclidean distance and rooted with strain CFBP5241 of *X. campestris *pv. *campestris*. Confidence on nodes was established using 1000 bootstrap replicates. Only bootstrap values above 50% are reported.

Based on the presence/absence matrix, clustering of strains from different pathovars could be linked in some cases to the host plant (Figure 2). Strains that were grouped were of the pathovar *vignicola*, which infect *Vigna unguiculata *and *V*. *sinensis*, and strains of GL2 of pathovar *phaseoli*, which infect *Phaseolus vulgaris*. The hosts of isolation of these strains belong to the same botanical family (*Fabaceae*) and are closely related [34]. Also clustered together were strains phylogenetically distant [30] and belonging to different species (CFBP5823 of *X. axonopodis *pv. *vasculorum *and CFBP5830, CFBP5831, and CFBP1215 of *X. vasicola *pv. *vasculorum*) but isolated from the same host, *Saccharum officinarum *(Figure 2).

### Positive selection acting on *X. axonopodis *and *X. campestris *genes encoding MCPs and adhesins

**Figure 3:**
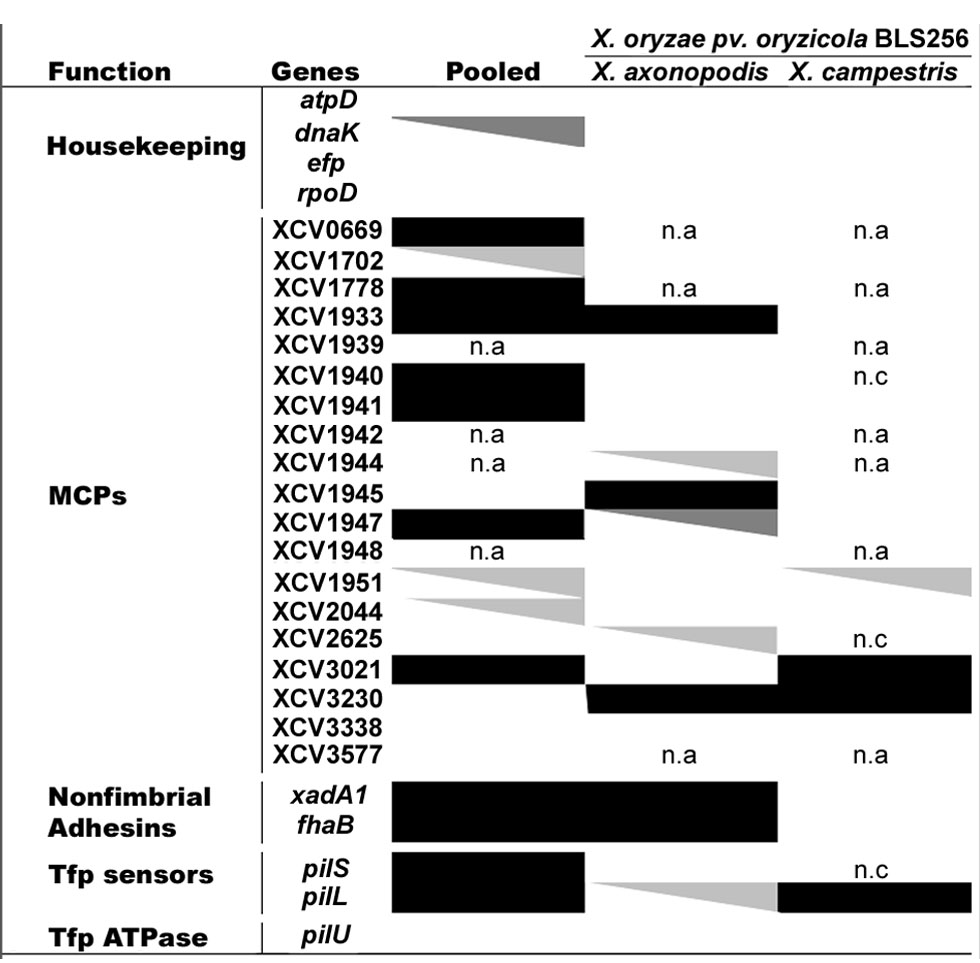
**MK test results for adaptive divergence on genes involved in sensing and adhesion**. Genes were extracted from sequenced genomes of three strains of *X. axonopodis *(*Xav*85-10, *Xac*306, and *Xap*CFBP4838) and three strains of *X. campestris *(*Xcc*ATCC33913, *Xcc*8004, and *Xcc*B100). The ratios of replacement and synonymous changes within a species were compared with the ratios of replacement and synonymous changes fixed between the species. Pooled refers to MK test done using both species, *X. axonopodis *and *X. campestris*. Using *X. oryzae *pv. *oryzicola *BLS256 as an outgroup served to ascertain which species has been affected by positive selection. A sequential Bonferroni correction for multiple tests was applied. Black squares correspond to high *p *values (*p *< 0.0034) and indicate robust evidence of positive selection before and after correction. Grey triangles indicate signals of positive selection, which are not considered after sequential Bonferroni correction: dark-grey and light grey triangles indicate *p *values ranging from 0.0034 to 0.01 and from 0.01 to 0.05, respectively. White squares correspond to non-significant *p *values (*p *> 0.05) and indicate no signal of positive selection. A non-available result (n.a) means that the orthologous sequences were lacking on the genome sequence. Lack of polymorphism did not allow test computation (n.c).

The McDonald-Kreitman (MK) tests for adaptive divergence done on 24 candidate genes (MCPs, adhesins, and Tfp sensors) and four housekeeping genes from three sequenced strains of *X. axonopodis *and three of *X. campestris *revealed that 13 of the 24 candidate genes showed robust signatures of adaptive divergence (Figure 3 and see additional file 3: Table A3 for the results of the MK tests). The four housekeeping genes did not display any signal of positive selection after Bonferroni correction. Significant signatures of adaptation were found in the divergence between *X. axonopodis *and *X. campestris *sequences on 11 genes (7 MCPs, 2 adhesins, and 2 Tfp sensors) of the 24 for which analysis was possible. Using *Xoc*BLS256 as an outgroup showed that diversifying selection preferentially affected the *X. axonopodis *clade.

**Figure 4:**
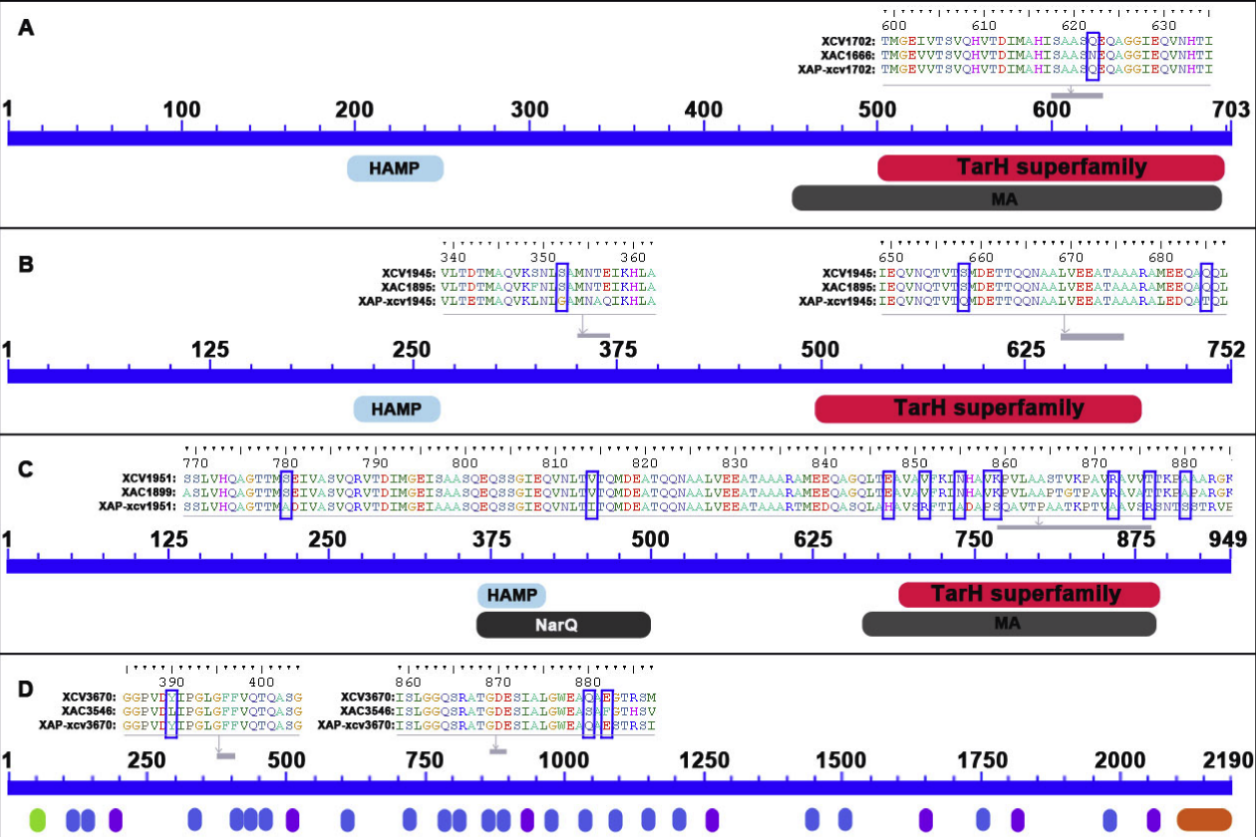
**Schematic representations of examples of sites under selection and conserved domains in MCPs (A, B, and C) and adhesins (D)**. In the upper parts of each panel the sequence alignments of proteins are presented for parts containing sites under selection. The orthologous sequences collected from genome sequences of *Xcv*85-10, *Xac*306, and *Xap*CFBP4834 were aligned using ClustalW according to the translated amino-acid sequences, manually performed using BIOEDIT. Sites that were found under selection are highlighted by green frames. In the lower parts of the panels, conserved domains are represented. HAMP: Histidine kinase, Adenylyl cyclase, Methyl-accepting protein, and Phosphatase domain. HAMP is a signalling domain that occurs in a wide-variety of signalling proteins. TarH superfamily: Taxis toward Aspartate and Related amino acids and Homologs. MA: Methyl-accepting chemotaxis-like domain (chemotaxis sensory transducer), thought to undergo reversible methylation in response to attractants or repellents during bacterial chemotaxis. NarQ: signal transduction histidine kinase, nitrate/nitrite-specific. Annotation bars coloured in grey refer to multi-domains that are excluded from domain-domain neighbouring. In the D panel, green, blue, purple, and orange ovals symbolised TAT signal, Hep-Hag motif, HIM motif and YadAlike, C-terminal domain, respectively.

Positive selection was also tested using the branch-site model implemented in PAML within *X. axonopodis *on candidate genes already tested with the MK test. The branch-site model allows detecting selection at a few codons (sites) on a specific lineage (branch). Strains of different pathovars in *X. axonopodis *(strains *Xav*85-10, *Xac*306 and *Xap*CFBP4834) occupy distinct habitats, exploit distinct niches, and cause different diseases on different host-plants (*Lycopersicon esculentum *and *Capsicum *sp., *Citrus *sp. and *Phaseolus vulgaris*, respectively). We hypothesised that these strains representing different pathovars would have undergone selection differently for correspondingly distinct adaptations. For each gene, three tests were applied, each considering a different foreground branch corresponding to each of the three pathovars. Results revealed that sets of genes under positive selection in *Xav*85-10, *Xac*306, and *Xap*CFBP4834 were distinct. Indeed in the case of pathovar *vesicatoria *(*Xav*85-10), the set of genes under selection included XCV1940 and XCV1942. Regarding pathovar *citri *(*Xac*306), the set included XCV1702, XCV1945, and *xadA1*. Finally, in pathovar *phaseoli *(*Xap*CFBP4834), positive selection was detected on genes XCV1945 and XCV1951. Interestingly, XCV1945 was found to be under positive selection on both pathovars *citri *and *phaseoli*. However, Bayes Empirical Bayes (BEB), which estimates the probabilities of each site on the foreground branch evolving under positive selection, identified different sites either in *Xac*306 or *Xap*CFBP4834 (Table 1). For each gene, from 1 to 10 sites were found under selection. Sixteen of the 22 sites under selection were in conserved domains in the proteins (Table 1 and Figure 4).

**Table 1:** Sites under positive selection in candidate genes.

Strain	**Target gene name**^**a**^	Residue	Residue position	***p*(ω > 1)**^**b**^	Interpro accession
*Xav*85-10	XCV1940	P	79	0,920	-
	XCV1942	I	48	0,910	-
	XCV1942	R	131	0,948	-
*Xac*306	XCV1702	Q	622	0,929	IPR004089
	XCV1945	D	59	0,909	-
	XCV1945	S	92	0,982	-
	*xadA1*	Y	390	0.903	-
	*xadA1*	Q	880	0.947	IPR008640
	*xadA1*	E	882	0.951	IPR008640
*Xap*CFBP4834	XCV1945	S	352	0,955	IPR004089
	XCV1945	S	658	0,954	IPR004089
	XCV1945	Q	685	0,945	IPR004089
	XCV1951	S	780	0,971	IPR004089
	XCV1951	V	814	0.924	IPR004089
	XCV1951	E	847	0.968	IPR004089
	XCV1951	V	851	0.984	IPR004089
	XCV1951	N	855	0.962	IPR004089
	XCV1951	V	858	0.975	IPR004089
	XCV1951	K	859	0.989	IPR004089
	XCV1951	R	872	0.982	IPR004089
	XCV1951	T	876	0.993	IPR004089
	XCV1951	A	880	0.982	IPR004089

Genes were extracted from genomic sequences of three strains belonging to three pathovars of *X. axonopodis (vesicatoria, citri *and *phaseoli)*. Sites were identified using Branch-site model A (model = 2 NSites = 2) compared to the null model with ω fixed to 1.

^a^: National Center for Biotechnology Information (NCBI) gene name. XCV indicates a gene in *X. axonopodis *pv. *vesicatoria *85-10 genome.

^b^: ω estimates the parameter *D*_n_/*D*_s _for each site and *p*(ω > 1) represents posterior probability of sites with ω > 1.

## Discussion

The early phases of host-colonisation are crucial for pathogenic bacteria [35]. However, little is known about the importance of early phases in determining host-specificity for plant pathogenic bacteria. Emphasis has been put on later phases of infection, as illustrated by the demonstration of the role of T3Es in host-specificity [1,2,36]. Host-recognition can be considered the first instance of host-pathogen interaction allowing bacteria to colonise. The importance of chemotaxis in plant-bacteria interactions has been clearly documented in some cases. For example the chemotactic mutant *Ralstonia solanacearum *is unable to colonise its host when inoculated into the soil, whereas it remains fully pathogenic when infiltrated inside plant tissues [10]. Thus evolutionary processes of host-specificity would also be driven by selective forces at the very first steps of host-colonisation. Our data show evidence of such adaptive processes for numerous genes involved in chemotactic attraction, environment sensing, and adhesion to surfaces. These mechanisms precede the infection of a plant by xanthomonads. The mechanisms facilitating plant penetration and thus allowing infection are chemotaxis, aerotaxis, and fast multiplication inside host tissue, which relies on adhesion.

According to MK-tests results, many candidate genes undergo positive selection during bacterial colonisation on plant tissue. Of the 24 genes common among repertoires of the studied pathovars (*vesicatoria*, *citri*, and *phaseoli*) of *X. axonopodis *and *X. campestris *pv. *campestris*, nearly half are subject to positive selection. This proportion is almost equal among the two gene families (MCPs and adhesins). The McDonald-Kreitman procedure tests for adaptive divergence between two species. The test is known to be robust to non-equilibrium demography [37,38] and to recombination [39]. Using a small sample size would reduce the power of the test. The lack of power to detect selection would increase the risk of false negatives. Thus our results are conservative and can not be interpreted as false positive. Charlesworth and Eyre-Walker [40] showed that about 50% of amino-acid substitutions surveyed in the enteric bacterial genomes were subject to adaptive evolution. Thus both recognition and adhesion should be considered as selective steps for bacterial colonisation. To our knowledge this is the first report of positive selection acting on MCPs in plant-pathogenic bacterium. Our results are consistent with those of Chen *et al. *[41] and Petersen *et al. *[42] showing that positive selection acts on genes encoding surface structures of *E. coli *cell. These genes encode regulators of LPS O-antigen chain length, putative adhesins that affect biofilm formation, ferrichrome-iron receptors, two outer membrane porins, and more [41,42]. In xanthomonads, the extracellular appendage of the Hrp pilus evolved under the constraint of positive selection likely to avoid recognition by plant defense surveillance systems [43].

Most sites that were found under selection in candidate genes by PAML analysis were located in conserved domains predicted to play a role in perception for MCPs and in adhesion for XadA1. Indeed, the Tar domain of chemoreceptors directly binds to aspartate and related amino acids [44]. The Hep-Hag motif is found in the passenger domain of adhesins [45]. This domain is known to contribute to the binding activity of invasins/agglutinins [23]. This result is another argument in favour of selection pressures acting on these genes in link with the ecological behaviour of the strains. Detection of positive selection using the branch-sites model implemented in PAML has some power limitations especially if only three sequences are used. Our results, however, should be considered conservative as they reduced the number of false positives.

Many bacteria assemble multifunctional proteic structures on their surfaces that serve for adhesion. This feature might be an adaptation to different environmental conditions and, in the case of pathogenic bacteria, to different hosts or host tissues [46]. In fact, adhesins are involved in various processes leading to host colonisation and transmission to seed by plant-pathogenic bacteria. For example, Tfp serves remarkably diverse functions, including twitching motility, cell to cell adhesion, and thus microcolony and biofilm formation [21]. Tfp is an important virulence factor for vascular and non vascular plant pathogens [47,48]. Moreover, Darsonval and colleagues [48] showed that PilA is involved not only in adhesion but also in transmission to seed, and the mutation of *pilA *in strain CFBP4834 of *X. axonopodis *pv. *phaseoli *GL *fuscans *leads to lower pathogenicity on bean (*P. vulgaris*). Additionally, YapH, an hemagglutinin, is required for adhesion to seed, leaves, and abiotic surfaces.

An interesting consequence of strong differential selection pressures by host is a specialisation at some early steps (i.e. chemotactic attraction) of host colonisation by xanthomonads. Character displacement at early stage of host colonisation should make infection more efficient by preventing competition for habitat between strains [49,50]. In fact, xanthomonads are known to be phenotypically very homogeneous except in pathogenicity. Lack of selective pressures in host colonisation would lead to colonisation by a wide range of incompatible strains. Colonisation of specialized pathovars would therefore be less successful. Indeed, resource allocation would be redirected in favour of competition detrimental to pathogenicity. Finally, such host-isolation could act as an ecological isolating barrier to limit recombination between differentially adapted pathogenic strains. As in eukaryotic organisms, ecological differences in bacteria are known to promote speciation [51]. Indeed, habitat sharing would allow recombination between strains that belong to different pathovars and that consequently produce strains that may reveal genetic incompatibilities.

This comparative analysis of repertoires of MCPs, STCRS, TBDTs, and adhesins provides useful insight into bacterial behaviour. First, the number of MCPs and more generally sensors is higher in *Xanthomonas *strains than in *E. coli *and *Salmonella *[52]. *E. coli *and *Salmonella *have only five MCPs whereas strains *Xav*85-10, *Xac*306, and *Xcc*ATCC339313 have about 20 MCPs; strain *Xoo*KACC10331 has only about 10 MCPs. The large numbers (14 in *Xav*85-10, 10 in *Xac*306, 8 in *Xcc*ATCC339313, and 7 in *Xoo*KACC10331) of MCPs and other sensors repeated in tandem are unusual in bacteria, suggesting a prominent role in the life style of *Xanthomonas *[17,31,32]. Second, repertoires of MCPs, STCRS, TBDTs, and adhesins differed among the majority of pathovars and genetic lineages belonging to the tested *Xanthomonas *spp. and displaying different host range. Repertoires of genes coding sensors and adhesins comprised core and variable gene suites. Some genes under study were not intra-specifically conserved and hence belong to the accessory genome. Repertoires of genes involved in attraction and adhesion may evolve by gene gain or loss, probably after duplication events. In the case of *Drosophila*, the size of repertoires of genes encoding olfactory and gustatory receptors varies through gene duplication, pseudogenization, and gene loss. The changes among species of *Drosophila *show that these receptors have changed during species divergence, and their evolution might reflect species' adaptation to their chemical environment [53]. Similarly, in xanthomonads, the variable set of sensors and adhesins may be involved in the recognition of specific components allowing strain adaptation to a particular set of hosts. Moreover, we identified signals of adaptive divergence have been identified on such genes of the variable set.

This study showed that among the same genus, *Xanthomonas*, the majority of pathovars and genetic lineages belonging to different species (*X. axonopodis*, *X. campestris*, *X. vasicola*, and *X. oryzae*) displayed different and unique repertoires of MCPs, STCRS, TBDTs, and adhesins while they displayed different host range. Note that the distribution of sensor and adhesin genes does not necessarily correlate with strain phylogeny. Indeed, bacteria as phylogenetically distant as *X. axonopodis *pv. *vasculorum *and *X. vasicola*. pv. *vasculorum *[30] share a common repertoire of sensor and adhesin genes (Figure 2) and a common host: sugarcane. This case illustrates the sharing of a common ecological niche (symptomatic host) by two phylogenetically distant bacteria.

Our results suggest that adaptation to host involves pathoadaptation but also asymptomatic colonisation steps. Indeed, several pathovars and genetic lineages shared the same repertoires whereas they are known to infect different crops. This means that they could share the same asymptomatic host range but develop symptoms on a restricted number of different host plants. This is the case for the pathovar *vignicola *and the genetic lineage GL2 of pathovar *phaseoli*. *X. axonopodis *pv. *phaseoli *GL2 and pv. *vignicola *strains may detect similar plant-originated molecules potentially conserved among their host plants. This hypothesis is supported by the fact that *X. axonopodis *pv. *phaseoli *GL2 and *vignicola *both infect legumes. *X. axonopodis *pv. *phaseoli *GL2 infects *Phaseolus *spp. and X. *vignicola *infects *Vigna unguiculata*; these legumes belong to the Milletioid clade and are phylogenetically closely related [33]. Interestingly, cross inoculations would provide insight on the ecological behaviours of these two pathogens (survival, colonisation and chemotaxis responses). The four genetic lineages (*fuscans*, GL1, GL2, GL3) of pathovar *phaseoli*, which all share a common host (*P. vulgaris*), present distinct repertoires of MCPs, STCRS, TBDTs, and adhesins. Genetic lineage *fuscans*, GL2, and GL3 are phylogenetically closely related and belong to rep-PCR group 9.6 whereas GL1 is distant and belongs to rep-PCR group 9.4 [2,30,54]. These four distinct genetic lineages have different T3E repertoires but clustered together on the dendrogram constructed on the matrix of presence/absence of T3Es genes, supporting the hypothesis of an adaptive pathological convergence on bean [2]. Our results suggest that, upstream of the invasive pathological stage, the four genetic lineages have different ecological behaviours. Colonisation does not necessarily lead to infection and then may not be under the same adaptive processes as host infection. We can speculate that each lineage of *X. axonopodis *pv. *phaseoli *can be found on different asymptomatic hosts.

Overall, our findings indicate that plant pathogenic bacteria belonging to different pathovars have evolved different set of genes allowing them to specifically detect favourable hosts on which they can settle. These results support a recent theory termed inverse-gene-for-gene in which infectiousness is determined by pathogen recognition of hosts signals and/or receptors [55]. This theory is an alternative to the gene-for-gene model in which the pathogen is recognized by the host., Here, in agreement with this theory we show that many pathogen genes involved in host recognition evolved under adaptive divergence. Such a selective pressure on genes encoding for recognition of specific hosts strongly accounts for coevolutionary dynamics where pathogens are always adapting their sensors in response to hosts changes in exudates and surface structures.

Plant pathogenic xanthomonads are associated with aerial parts of plants. They are not usually encountered in other environments. Plant pathogenic pseudomonads colonise non-host habitats such as snow or water [56]. Our attempts to isolate xanthomonads from such environments were, however, unsuccessful (our unpublished data). Saprophytic survival of xanthomonads in soil is very poor. Apart from their primary host, many xanthomonads can survive for long periods in association with weeds that grow naturally in crops. It is not yet known which weeds are susceptible to colonisation by each plant pathogenic xanthomonad. We refer in our study to the main crop contaminated by each pathovar, which certainly represents the major opportunity for bacterial multiplication.

## Conclusions

This study showed that the majority of the tested pathovars belonging to different species of *Xanthomonas *(*X. axonopodis*, *X. campestris*, *X. vasicola*, and *X. oryzae*) displayed unique repertoires of genes coding proteins involved in sensing (MCPs, STCRS, TBDTs) and adhesion. Our data show evidence of adaptive processes for numerous genes involved in chemotactic attraction, environment sensing, and adhesion to surfaces. Most sites that were found under selection in candidate genes were located in conserved domains predicted to play a role in perception or in adhesion. Xanthomonads are plant-associated bacteria and are not known to efficiently colonise other environments. Hence, we argue that the gene evolution we observed may reflect pathovar adaptation to the host-plant environment.

The molecular variation of genes involved in host recognition and adhesion to host tissues clearly shows adaptation begins at the very first steps of host colonisation. We suggest that such adaptive divergence at early phases of host colonisation would act as an ecological isolating barrier to promote speciation. However, such a process could have appeared after isolation by genes involved in infection, like T3Es. In this case, adaptation would be a consequence rather than a cause. Despite their high evolutionary relevance, the respective roles of host recognition and host infection in promoting ecological reproductive barriers remain to be elucidated.

## Methods

The xanthomonad strains used in this study were named following the nomenclature proposed by Vauterin *et al. *[27,57] and are presented in Additional file 2. Genes encoding MCPs, STCRS, TBDTs, and adhesin-related genes were selected from sequenced genomes of *Xav*85-10, *Xac*306, *Xcc*ATCC33913, and *Xoo*KACC10331 http://www.ncbi.nlm.nih.gov. These genes were identified according to their function-based assignment in the NCBI website to cell motily (COG N), signal transduction mechanisms (COG T), and inorganic ion transport and metabolism (COG P) classes and according to Thieme *et al. *[32] for fimbrial and nonfimbrial adhesin genes. Using Blastx analysis with default parameters [58], we identified orthologous genes (more than 80% identity on more than 80% of the length of the sequence) in each of the four reference genomes. Hence, a list containing 320 genes involved in sensing, chemotaxis, motility, and adhesion was established (See Additional file 1: Table A1). This list was refined by analysing the functional-domain composition of the genes. We selected genes encoding MCPs containing periplasmic domains (*e.g *TAR, PRK09793, PAS and HAMP domains). Regarding genes encoding TBDTs and STCRS, we selected receptor and sensor parts, respectively, based on the gene description and gave priority to genes identified in only one of the four sequenced strains. We discarded genes that encoded cytoplasmic proteins such as chemotactic proteins (CheA-Z) and genes encoding flagellar components characterised by a very low polymorphism of presence or absence and sequence conservation.

Primer design was based on the alignment of orthologous sequences collected from the four genomes in conserved fragments, taking care to avoid amplification of other genes that share domains with the target. Then, sets of primers were validated both by *in silico *specific gene amplifications (Amplify software version 3.1.4) and by specific gene amplification using PCR with genomic DNA extracted from the four sequenced genomes previously mentioned. PCR reactions were prepared as described previously [2]). To characterise repertoires, PCRs were performed using one set of extracted genomic DNA (1 ng) and two sets of boiled bacterial cells (3 × 10^8 ^cfu ml^-1^) per strain. Preliminary dot blot hybridisations were done to validate PCR results. We designed probes for specific (XCC0324) and variable (XCV2103 and XAC1816) genes. Hybridisations were performed as described previously [2] on extracted DNA (250 ng) from reference strains. Positive signals were obtained for all strains, in contradiction with the results expected based on bioinformatic analysis of presence/absence on the sequenced-genomes from the same strains. These discrepancies can be explained by the structure of these modular genes, which share motifs in functional domains (*e.g*. HAMP, hemagglutinin, or autotransporter domains) and consequently have many sequence similarities. As dot blot could not be used to confirm the absence of signal we designed, whenever possible, another set of primers for each non-ubiquitous gene. Generally, two sets of primers were used for each gene on three batches of DNA (See Additional file 4: Table A4 for the sequences of primers). Finally, a fourth set for result validation was obtained using the draft genome sequence of *X. axonopodis *pv. *phaseoli *GL *fuscans *strain CFBP4834 (our unpublished data). Every PCR result on the DNA of this strain was confirmed by BLAST analysis [56].

Based on the presence/absence matrix of MCPs, STCRS, TBDTs, and adhesin genes obtained by PCRs for each of the 173 strains of *Xanthomonas *sp., we constructed a dendrogram using Euclidean distance and Neighbour-Joining method, which was visualised using PAST version 1.90 software [59].

Tests for positive selection were performed on orthologous sequences collected from the genome sequences of *Xav*85-10, *Xac*306 and *Xap*CFBP4834, *Xcc*ATCC33913, *Xcc*8004 and *Xcc*B100, and *Xoc*BLS256. Twenty four candidate genes, including 19 MCPs genes and 5 adhesin-related genes, were analysed together with 4 housekeeping genes. The nucleotide sequences were aligned using ClustalW [60] according to the translated amino-acid alignment in order to keep the codon structure of the coding sequences. Adjustments on multiple alignments were manually performed using BIOEDIT version 7.0.9.0 program [61]. Annotation of domains was performed by using the Conserved Domain Database available [62] in NCBI web site. The DNASP software package [63] was used to perform several tests for positive selection using the MK test [64]. The MK test is a simple method to contrast the patterns of within-species polymorphism and between-species divergence at synonymous and non-synonymous (replacement) sites in the encoding region of a gene. The MK test evaluates whether an excess of replacement mutations versus synonymous mutations had been fixed between the two species compared with replacement and synonymous polymorphisms within each species. A significant ratio of fixed replacement to fixed synonymous mutations leads to rejecting the neutral mutation hypothesis and indicates adaptive fixation of selectively advantageous mutations. Three MK test conditions were applied (i) to detect adaptive divergence signals between two species (*X. axonopodis *vs *X. campestris*) and (ii) to ascertain which species has been affected by positive selection by assigning the fixed replacement changes to *X. axonopodis *and/or *X. campestris *using *X. oryzae *pv. *oryzicola *(*Xoc*) strain BLS256 as outgroup strain. The significance of MK results was established by the Fisher exact test. Bonferroni correction for multiple tests [65] was hand-computed and applied on MK test results. Testing for positive selection acting at a specific locus on a particular pathovar (*i.e. *branch) was performed using the branch-site model A of Yang and Nielsen [66] implemented in the CODEML program of the PAML package (version 3.14) [66,67]. Testing was performed on each gene previously analysed for adaptive divergence. Three sequenced-genome strains of different pathovars in *X. axonopodis *(pvs. *vesicatoria*, *citri*, and *phaseoli*) were used. For each gene, three tests were done by labelling one branch as foreground at a time. Model selection was performed using likelihood ratio test. In contrast to other methods of selection detection, the Branch-site model allows for small numbers of taxa.

## Authors' contributions

NMH design and performed the experiments, analyzed the data and drafted the manuscript. AD participated in the design of the study, in the construction of gene repertoires, in the data analyses and in the writing. SP and KD participated in the construction of gene repertoires. SF and VB performed sequencing of CFBP4834 genome. CM participated in the design of the study and revised the manuscript. CL analyzed the data and wrote the paper. MAJ conceived the study, design the experiments, analyzed the data and wrote the paper. All authors read and approved the final manuscript.

## Supplementary Material

Additional file 1 **Table A1 Genome mining: identification of genes involved in sensing and adhesion in xanthomonads**. Genes were extracted from 4 complete sequenced genomes of xanthomonads available in the NCBI website Click here for file

Additional file 2 **Table A2 List of bacterial strains used in this study and their repertoires of candidate genes**. Strains belonged to 25 pathovars of *Xanthomonas *spp. MCPs, STCRS, and TBDTs are involved in sensing while Tfp sensors and adhesins are involved in adhesion. Presence (black square) or absence (white square) of an orthologous sequence was determined by at least three independent PCR reactions Click here for file

Additional file 3 **Table A3 Genes under positive selection**. Results of the MK tests for positive selection on 4 genes encoding housekeeping genes, 19 genes encoding MCPs, and 5 adhesin-related genes Click here for file

Additional file 4 **Table A4 Sequences of pairs of primers used to amplify candidate genes**. The annealing temperature of the PCRs and the lengths of the amplified fragments are also indicated. Click here for file
