# Supplementary material for: Sensing and adhesion are adaptive functions in the plant pathogenic xanthomonads
Source: BMC Evol Biol. 2011 Mar 11;11:67. doi: 10.1186/1471-2148-11-67 (PMC3063832; doi:10.1186/1471-2148-11-67)
Supplement: Additional file 3 — Table A3 Genes under positive selection. Results of the MK tests for positive selection on 4 genes encoding housekeeping genes, 19 genes encoding MCPs, and 5 adhesin-related genes [file 1471-2148-11-67-S3.DOC]

**Table A3 Results of the MK tests for positive selection on genes encoding MCPs and adhesins.** Four housekeeping genes (*atpD*, *dnaK*, *efp* and *rpoD*) were used as controls. The cell entries represent the observed numbers of polymorphic synonymous sites (P_s_), polymorphic nonsynonymous sites (P_n_), divergent synonymous sites (D_s_), and divergent nonsynonymous sites (D_n_). A significant excess of nonsynonymous divergence (D_n_/D_s_ > P_n_/P_s_) leads to rejecting the neutral mutation hypothesis and is interpreted as a signal of positive selection. Mk tests were performed (i) on pooled sequences from 6 strains (*Xav*85-10, *Xac*306 and *Xap*CFBP4834, *Xcc*ATCC33913, *Xcc*8004 and *Xcc*B100) belonging to two species (*X. axonopodis* and *X. campestris*) to detect adaptive divergence signals, and (ii) by assigning the fixed replacement changes to the pooled sequences from *X. axonopodis* (iia) or *X. campestris* (iib) using *X. oryzae* pv. *oryzicola* (*Xoc*) strain BLS256 as outgroup to ascertain which species has been affected by positive selection.

|  | P_s_ | P_n_ | D_s_ | D_n_ | *p* value  ^a^ | *G* value  ^b^ |
| --- | --- | --- | --- | --- | --- | --- |
| ***atpD*** |  |  |  |  |  |  |
| (i) pooled | 76 | 9 | 23 | 2 | 1.00 | 0.15 |
| (iia) *X. axonopodis vs Xoc*BLS256 | 64 | 7 | 19 | 2 | 1.00 | 0.00 |
| (iib) *X. campestris* vs *Xoc*BLS256 | 18 | 2 | 44 | 6 | 1.00 | 0.06 |
| ***dnaK*** |  |  |  |  |  |  |
| (i) pooled | 73 | 1 | 47 | 8 | 4.71 × 10^-3^ ** | 9.07 ** |
| (iia) *X. axonopodis vs Xoc*BLS256 | 69 | 1 | 36 | 3 | 0.13 | 2.65 |
| (iib) *X. campestris* vs *Xoc*BLS256 | 4 | 0 | 81 | 11 | 1.00 | - ^c^ |
| ***efp*** |  |  |  |  |  |  |
| (i) pooled | 14 | 1 | 17 | 6 | 0.21 | 2.56 |
| (iia) *X. axonopodis vs Xoc*BLS256 | 12 | 1 | 14 | 2 | 1.00 | 0.18 |
| (iib) *X. campestris* vs *Xoc*BLS256 | 2 | 0 | 31 | 6 | 1.00 | - |
| ***rpoD*** |  |  |  |  |  |  |
| (i) pooled | 90 | 14 | 41 | 5 | 0.79 | 0.20 |
| (iia) *X. axonopodis vs Xoc*BLS256 | 52 | 2 | 39 | 6 | 0.14 | 3.14 |
| (iib) *X. campestris* vs *Xoc*BLS256 | 69 | 13 | 69 | 5 | 8.14 × 10^-3^ ** | 8.27 ** |
| **XCV0669** |  |  |  |  |  |  |
| (i) pooled | 107 | 12 | 153 | 47 | 2.76 × 10^-3^ ** | 9.58 ** |
| (iia) *X. axonopodis vs Xoc*BLS256 | / ^d^ | / | / | / | / | / |
| (iib) *X. campestris* vs *Xoc*BLS256 | / | / | / | / | / | / |
| **XCV1702** |  |  |  |  |  |  |
| (i) pooled | 102 | 42 | 226 | 159 | 0.01 * | 6.71 ** |
| (iia) *X. axonopodis vs Xoc*BLS256 | 99 | 40 | 99 | 51 | 0.36 | 0.91 |
| (iib) *X. campestris* vs *Xoc*BLS256 | 3 | 2 | 246 | 185 | 1.00 | 0.00 |
| **XCV1778** |  |  |  |  |  |  |
| (i) pooled | 86 | 24 | 217 | 138 | 8.95 × 10^-4^ *** | 11.37 *** |
| (iia) *X. axonopodis vs Xoc*BLS256 | / | / | / | / | / | / |
| (iib) *X. campestris* vs *Xoc*BLS256 | / | / | / | / | / | / |
| **XCV1933** |  |  |  |  |  |  |
| (i) pooled | 98 | 30 | 139 | 95 | 1.16 × 10^-3^ ** | 11.15 |
| (iia) *X. axonopodis vs Xoc*BLS256 | 62 | 2 | 82 | 28 | 1.08 × 10^-4^ *** | 17.37 *** |
| (iib) *X. campestris* vs *Xoc*BLS256 | 39 | 28 | 163 | 98 | 0.57 | 0.40 |
| **XCV1939** |  |  |  |  |  |  |
| (i) pooled | / | / | / | / | / | / |
| (iia) *X. axonopodis vs Xoc*BLS256 | 106 | 45 | 85 | 26 | 0.26 | 1.33 |
| (iib) *X. campestris* vs *Xoc*BLS256 | / | / | / | / | / | / |
| **XCV1940** |  |  |  |  |  |  |
| (i) pooled | 88 | 35 | 140 | 139 | 0.78 × 10^-4^ *** | 16.33 *** |
| (iia) *X. axonopodis vs Xoc*BLS256 | 88 | 35 | 68 | 30 | 0.77 | 0.12 |
| (iib) *X. campestris* vs *Xoc*BLS256 | 0 | 0 | 170 | 158 | - | - |
| **XCV1941** |  |  |  |  |  |  |
| (i) pooled | 83 | 24 | 154 | 96 | 3.36 × 10^-3^ ** | 8.94 ** |
| (iia) *X. axonopodis vs Xoc*BLS256 | 91 | 29 | 93 | 37 | 0.47 | 0.59 |
| (iib) *X. campestris* vs *Xoc*BLS256 | 3 | 0 | 196 | 105 | 0.55 | - |
| **XCV1942** |  |  |  |  |  |  |
| (i) pooled | / | / | / | / | / | / |
| (iia) *X. axonopodis vs Xoc*BLS256 | 102 | 66 | 69 | 30 | 0.15 | 2.21 |
| (iib) *X. campestris* vs *Xoc*BLS256 | / | / | / | / | / | / |
| **XCV1944** |  |  |  |  |  |  |
| (i) pooled | / | / | / | / | / | / |
| (iia) *X. axonopodis vs Xoc*BLS256 | 77 | 63 | 72 | 95 | 0.04 * | 4.32 * |
| (iib) *X. campestris* vs *Xoc*BLS256 | / | / | / | / | / | / |
| **XCV1945** |  |  |  |  |  |  |
| (i) pooled | 449 | 259 | 8 | 20 | 0.43 | 0.77 |
| (iia) *X. axonopodis vs Xoc*BLS256 | 276 | 110 | 122 | 96 | 1.66 × 10^-4^ *** | 14.77 *** |
| (iib) *X. campestris* vs *Xoc*BLS256 | 224 | 136 | 151 | 112 | 0.25 | 1.46 |
| **XCV1947** |  |  |  |  |  |  |
| (i) pooled | 107 | 25 | 136 | 89 | 0.57 × 10^-4^ *** | 17.07 *** |
| (iia) *X. axonopodis vs Xoc*BLS256 | 100 | 22 | 117 | 58 | 5.06 × 10^-3^ ** | 8.62 ** |
| (iib) *X. campestris* vs *Xoc*BLS256 | 7 | 3 | 204 | 103 | 1.00 | 0.06 |
| **XCV1948** |  |  |  |  |  |  |
| (i) pooled | / | / | / | / | / | / |
| (iia) *X. axonopodis vs Xoc*BLS256 | 55 | 11 | 91 | 22 | 0.69 | 0.22 |
| (iib) *X. campestris* vs *Xoc*BLS256 | / | / | / | / | / | / |
| **XCV1951** |  |  |  |  |  |  |
| (i) pooled | 157 | 63 | 228 | 140 | 0.02 * | 5.46 * |
| (iia) *X. axonopodis vs Xoc*BLS256 | 132 | 58 | 136 | 43 | 0.20 | 1.97 |
| (iib) *X. campestris* vs *Xoc*BLS256 | 28 | 7 | 315 | 198 | 0.03 * | 5.28 * |
| **XCV2044** |  |  |  |  |  |  |
| (i) pooled | 32 | 0 | 43 | 9 | 0.01 * | - |
| (iia) *X. axonopodis vs Xoc*BLS256 | 30 | 0 | 36 | 2 | 0.50 | - |
| (iib) *X. campestris* vs *Xoc*BLS256 | 2 | 0 | 67 | 11 | 1.00 | - |
| **XCV2625** |  |  |  |  |  |  |
| (i) pooled | 59 | 7 | 134 | 34 | 0.09 | 3.30 |
| (iia) *X. axonopodis vs Xoc*BLS256 | 59 | 7 | 52 | 21 | 0.01 * | 7.42 ** |
| (iib) *X. campestris* vs *Xoc*BLS256 | 0 | 0 | 150 | 44 | - | - |
| **XCV3021** |  |  |  |  |  |  |
| (i) pooled | 107 | 15 | 108 | 52 | 0.69 × 10^-4^ *** | 16.49 *** |
| (iia) *X. axonopodis vs Xoc*BLS256 | 80 | 18 | 103 | 15 | 0.26 | 1.32 |
| (iib) *X. campestris* vs *Xoc*BLS256 | 33 | 1 | 158 | 61 | 5.62× 10^-4^ *** | 13.63 *** |
| **XCV3230** |  |  |  |  |  |  |
| (i) pooled | 75 | 24 | 102 | 2 | 3.93× 10^-4^ *** | 14.13 *** |
| (iia) *X. axonopodis vs Xoc*BLS256 | 54 | 0 | 65 | 29 | 0.23 × 10^-4^ *** | 19.43 *** |
| (iib) *X. campestris* vs *Xoc*BLS256 | 23 | 0 | 127 | 43 | 2.77 × 10^-3^ ** | - |
| **XCV3338** |  |  |  |  |  |  |
| (i) pooled | 75 | 22 | 118 | 62 | 0.06 | 4.25 * |
| (iia) *X. axonopodis vs Xoc*BLS256 | 66 | 20 | 86 | 37 | 0.35 | 1.20 |
| (iib) *X. campestris* vs *Xoc*BLS256 | 10 | 3 | 165 | 84 | 0.56 | 0.67 |
| **XCV3577** |  |  |  |  |  |  |
| (i) pooled | 82 | 14 | 150 | 38 | 0.27 | 1.39 |
| (iia) *X. axonopodis vs Xoc*BLS256 | / | / | / | / | / | / |
| (iib) *X. campestris* vs *Xoc*BLS256 | / | / | / | / | / | / |
| ***xadA1*** |  |  |  |  |  |  |
| (i) pooled | 156 | 26 | 252 | 107 | 0.55 × 10^-4^ *** | 16.76 *** |
| (iia) *X. axonopodis vs Xoc*BLS256 | 141 | 22 | 156 | 105 | 0.00× 10^-4^ *** | 36.87 *** |
| (iib) *X. campestris* vs *Xoc*BLS256 | 15 | 4 | 315 | 184 | 0.22 | 2.15 |
| ***fhaB*** |  |  |  |  |  |  |
| (i) pooled | 536 | 263 | 1245 | 900 | 0.08 × 10^-4^ *** | 20.22 *** |
| (iia) *X. axonopodis vs Xoc*BLS256 | 499 | 238 | 1443 | 3191 | 0.00 × 10^-4^ *** | 353.32 *** |
| (iib) *X. campestris* vs *Xoc*BLS256 | 0 | 0 | 1577 | 3246 | - | - |
| ***pilS*** |  |  |  |  |  |  |
| (i) pooled | 64 | 9 | 122 | 53 | 2.334× 10^-3^ ** | 9.76 ** |
| (iia) *X. axonopodis vs Xoc*BLS256 | 51 | 8 | 105 | 28 | 0.24 | 1.58 |
| (iib) *X. campestris* vs *Xoc*BLS256 | 13 | 1 | 155 | 53 | 0.20 | 3.02 |
| ***pilL*** |  |  |  |  |  |  |
| (i) pooled | 232 | 61 | 406 | 246 | 0.00 × 10^-4^ *** | 27.65 *** |
| (iia) *X. axonopodis vs Xoc*BLS256 | 156 | 45 | 264 | 126 | 0.01 * | 6.52 * |
| (iib) *X. campestris* vs *Xoc*BLS256 | 79 | 17 | 485 | 277 | 2.27 × 10^-4^ *** | 14.51 *** |
| ***pilU*** |  |  |  |  |  |  |
| (i) pooled | 37 | 2 | 60 | 3 | 1.00 | 0.01 |
| (iia) *X. axonopodis vs Xoc*BLS256 | 36 | 1 | 41 | 6 | 0.13 | 3.09 |
| (iib) *X. campestris* vs *Xoc*BLS256 | 1 | 1 | 71 | 8 | 0.21 | 1.94 |

^a^: The significance of MK results was established by the Fisher's exact test (*: 0.01<*p*<0.05; **: 0.001< *p* <0.01; ***: *p* <0.001).

^b^: Value of the log-likelihood ratio test and significance of the associated probability (*: 0.01<*p*<0.05; **: 0.001< *p* <0.01; ***: *p* <0.001).

^c^: cannot be calculated due to an absence of polymorphism.

^d^: unavailable data due to the absence of the gene in the sequences of *X. campestris*.
