# Supplementary material for: Sensing and adhesion are adaptive functions in the plant pathogenic xanthomonads
Source: BMC Evol Biol. 2011 Mar 11;11:67. doi: 10.1186/1471-2148-11-67 (PMC3063832; doi:10.1186/1471-2148-11-67)
Supplement: Additional file 4 — Table A4 Sequences of pairs of primers used to amplify candidate genes. The annealing temperature of the PCRs and the lengths of the amplified fragments are also indicated. [file 1471-2148-11-67-S4.DOC]

**Table A4 Sequences of pairs of primers used to amplify candidate genes**. The annealing temperature of the PCRs and the lengths of the amplified fragments are also indicated.

| **First pair of primers** | | | | **Second pair of primers** | | | |
| --- | --- | --- | --- | --- | --- | --- | --- |
| Primers' name | **Reverse and Forward Primers** | **T °C** | **Fragment size (bp)** | Primers' name | **Reverse and Forward Primers** | **T °C** | **Fragment size (bp)** |
| XCV1940-Rev1 | GCCAGATCAGCTTCCGCATGGATG | 60 | 417 | Ubiquitous gene, no second pair of primers was designed | | | |
| XCV1940-Fwd1 | AGGTCGCCGGCTGCCATCGACT | 60 |  |  |  |  |  |
| XCV3230-Rev1 | TCGGCAGCGTCAGCACCAGCTTCTGG | 60 | 871 | Ubiquitous gene, no second pair of primers was designed | | | |
| XCV3230-Fwd1 | GCGCGTCTCGCGCACCGAACCGAA | 60 |  |  |  |  |  |
| XCV3021-Rev1 | GCGGGCTGCGCAAGGTGCAGAG | 65 | 964 | Ubiquitous gene, no second pair of primers was designed | | | |
| XCV3021-Fwd1 | CATGGTGGTGTTGACGTAGCGCTGCAT | 65 |  |  |  |  |  |
| XCV2044-Rev1 | AACGAACTGCGTACGTTCGTCAGCAAC | 60 | 738 | Ubiquitous gene, no second pair of primers was designed | | | |
| XCV2044-Fwd1 | GCATGTCCTCGAACTGCAGGGCA | 60 |  |  |  |  |  |
| XCV3338-Rev1 | GCTGGACGATCTGGACACCATGATCC | 60 | 338 | XCV3338-Rev2 | ACACCGATGATCTCGCCGATCTTGCGCGAGGC | 65 | 839 |
| XCV3338-Fwd1 | ACCTGCGCGGTGACGTCCATCCAC | 60 |  | XCV3338-Fwd2 | ATGCTCAAGCTGATCGAGCCGGATGTGCAGAGCTT | 65 |  |
| XCV2625-Rev1 | CTGGCGCAATTCGATCGCGCCTGGCA | 60 | 751 | XCV2625-Rev2 | AGCCGGAAGAACGCCATGCTCTGCTGCAG | 65 | 1285 |
| XCV2625-Fwd1 | GCGATGTCGTCGATGATGCCGATCTTC | 60 |  | XCV2625-Fwd2 | TGGCCAGCAGCGCAGAAGAGCGCCAGTC | 65 |  |
| XCV1933-Rev1 | GCTGGCGTTCGGCATCGTGCTGCTGAT | 65 | 1142 | XCV1933-Rev2 | ACGCTGGCGACGATTTCGGCCACGGTG | 65 | 2009 |
| XCV1933-Fwd1 | ACTTCGATGGTGCGGTCGCGCCATTC | 65 |  | XCV1933-Fwd2 | ATGCAATGGATCAACAATCTGAAACTGATGCC | 65 |  |
| XCV1778-Rev1 | GTGATGGCGGTCATCGAGTTTTCGC | 65 | 1020 | XCV1778-Rev2 | CAAGGCTACGCACTTCGGTCGCCACCAC | 65 | 1483 |
| XCV1778-Fwd1 | TCACCTCGATGATCTGACCGATGCGGT | 65 |  | XCV1778-Fwd2 | TTCGATCTGGACGGTCGCATTCTGGATGC | 65 |  |
| XCV1702-Rev1 | GTCAACAGCGTCAAGCAACGCTACG | 65 | 1352 | XCV1702-Rev2 | TTGGTCTGGAAGGCGATGCCGTCGATGAC | 65 | 1296 |
| XCV1702-Fwd1 | TTGACGGTGGAGGTCAGCTCTTCCATC | 65 |  | XCV1702-Fwd2 | CGAGCAGCGCACCCTGCCGCTGATC | 65 |  |
| XCV0669-Rev1 | ATTACTTCTTCATCGTCGACCCGCAGATGC | 60 | 1623 | XCV0669-Rev2 | GTCATTTCGTCCAGCTGCATCACCGTGGTGCT | 65 | 1586 |
| XCV0669-Fwd1 | TCTCGGCCATGATGTCGGTCACGCGCTT | 60 |  | XCV0669-Fwd2 | ATCAAGACCGCCATCGATAGCGGCGA | 65 |  |
| XCV1952-Rev1 | CGCTTCTCCGCGCAGACCAAGGTGAT | 65 | 857 | XCV1952-Rev2 | GAAGAGACGATCTCGGCCATGGTCTTGCC | 65 | 1334 |
| XCV1952-Fwd1 | GTCGATGACGCTGATGATCTCGGCGATCTT | 65 |  | XCV1952-Fwd2 | GACACCCAATCGCAGGCGCTGGTTTTCAC | 65 |  |
| XCV1941-Rev1 | TTCCGCCATGAGCTGGACAAGGCGA | 65 | 1538 | No other sets of primers were specific | | | |
| XCV1941-Fwd1 | CACCAGCTGGTTGCCGTGTTCGAC | 65 |  |  |  |  |  |
| XCV1938-Rev1 | AGCACCACCAAACTGGGCAAGGAC | 65 | 400 | Ubiquitous gene, no second pair of primers was designed | | | |
| XCV1938-Fwd1 | GGGCGGGTGACCATCTTGCG | 65 |  |  |  |  |  |
| XCV1951-Rev1 | GAGTGGGTCGGCATGGGCACCTTGTG | 65 | 2022 | XCV1951-Rev2 | GCCGACCACCGCATCGGCACTGAAGC | 65 | 1213 |
| XCV1951-Fwd1 | TGCTCACCGAATCGTCGATCAGGTGCTTGA | 65 |  | XCV1951-Fwd2 | CTACCAGGCCGAGCAGATCGGCAGCGA | 65 |  |
| XCV1954-Rev1 | TCGCGATCATCGTCGAAGTCAATAACCG | 60 | 400 | XCV1954-Rev2 | ATGAAATTCCTGCATTCCATGACCGTCGGGC | 65 | 1337 |
| XCV1954-Fwd1 | ATTCTGCTGGTTCTGCAATTCCACCG | 60 |  | XCV1954-Fwd2 | CGCCCGACGATGGTGGAGAGCTGT | 65 |  |
| XCV1947-Fwd1 | GGACGAGGTTTCGATGCCGGACATGGTGGTGACCACCT | 65 | 1353 | XCV1947-Fwd2 | TCGATGATGGAAGTCAGCGACCGCAACC | 65 | 434 |
| XCV1947-Rev1 | CTTGCGCATGGATGAAATCCAGGCCAAGTACGCC | 65 |  | XCV1947-Rev2 | ACGAGGTTTCGATGCCGGACATGGTGG | 65 |  |
| XCV1942-Rev1 | GGATCACCCGCAGCCTGACCCAGCCCTTG | 65 | 1217 | XCV1942-Rev2 | TCCACCAGCGCGGCGTTCTGCTGGG | 65 | 1976 |
| XCV1942-Fwd1 | CGCTGTGCACCAGCAGCGAGCCT | 65 |  | XCV1942-Fwd2 | CGCCTTTCCGCTGCGTTCGGCTTCCTG | 65 |  |
| XCV1939-Rev1 | ATGACCAAGATCCGCCTCTCCAACGACAT | 65 | 774 | XCV1939-Rev2 | ATCTGCGTGACGGTGAGGTTGACCTGTTCGAT | 65 | 1976 |
| XCV1939-Fwd1 | TGTGGCTGTCGACCAGCGCATTGGTGT | 65 |  | XCV1939-Fwd2 | ATGACATCTCTTTTACTGCGTTTCAATGTAGGGCC | 65 |  |
| XCV1944-Rev1 | CCGACAACCCGGTGCAGCAGGAACG | 60 | 524 | XCV1944-Rev2 | CTGCCAGCTGGTCTCGACGGTCGTCGAT | 65 | 2061 |
| XCV1944-Fwd1 | CCTGATCGTGCCGGCGCTTCATTTCCT | 60 |  | XCV1944-Fwd2 | ATGTCCATTGCCACGGTCAACATCGAGAC | 65 |  |
| XCV3261-Rev1 | CCTGATCACCATGTCCAATGCGCGTTATGA | 65 | 507 | No other sets of primers were specific | | | |
| XCV3261-Fwd1 | AGCGATGCCGCCGCTGGCCAGGAT | 65 |  |  |  |  |  |
| XCV1955-Rev1 | ACCGAGTTGTACGACGAGGTCAAACTGAAACTG | 65 | 1190 | XCV1955-Rev2 | TGGTGCTGGTCGTGCTGCTGGCTGCATTT | 65 | 1613 |
| XCV1955-Fwd1 | GGCGAGTTGGTTGGCCTGGCGCGCAT | 65 |  | XCV1955-Fwd2 | ACCGGCCGATACAGGGGCGCGCTT | 65 |  |
| XAC3768-Rev1 | TGATCGCCCACCTGCTGGAGAGCC | 65 | 2134 | XAC3768-Rev2 | TGATCGCCCACCTGCTCGAGAGCC | 65 | 573 |
| XAC3768-Fwd1 | TCAGGCGACGTGTTTTTCCAGGTGCTG | 65 |  | XAC3768-Fwd2 | TGGCGGTGACCGAGCTGTCCACCG | 65 |  |
| XAC3271-Fwd1 | CCGAGGCGGGGCATGGCGCCAAGCTGGTGGAGATAG | 65 | 454 | XAC3271-Fwd2 | TCAGAGCCGCGGCCAGCGAAAC | 65 | 461 |
| XAC3271-Rev1 | GAGCCGCGGCCAGCGAACCCCTGGTGCGCCACTGCGT | 65 |  | XAC3271-Rev2 | GTGACCGAGGCGGGGCATGG | 65 |  |
| XCC0324-Rev1 | ATGCACTTCGGCGTCTTCGTGATCCTG | 65 | 1770 | XCC0324-Rev2 | ATGCACTTCGGCGTCTTCGTGATCCTGG | 65 | 1320 |
| XCC0324-Fwd1 | TGGGCGCTGCGTCTTCCACAAAAAGG | 65 |  | XCC0324-Fwd2 | GGCCAACCCATCGATGACGGCGATGATG | 65 |  |
| XCC0276-Rev1 | GTGGCGCAGGCATCGGCGAGCGAA | 65 | 1890 | XCC0276-Rev2 | GTGGCGCAGGCATCGGCGAGCGAA | 65 | 645 |
| XCC0276-Fwd1 | TCGCACGTGCCGCGGCCGAGGCTT | 65 |  | XCC0276-Fwd2 | GGAAACATCGGCGAGCGTGCTGACCAC | 65 |  |
| XCV2152-Fwd1 | TGACACCCTTATGGATGCGCTTCATCCTG | 65 | 1996 | No other sets of primers were specific | | | |
| XCV2152-Rev1 | caattgcagcaacgctccgggcaaaaac | 65 |  |  |  |  |  |
| XCV2155-Fwd1 | AACTCGGACTGGCGCTGTACCACCAAG | 65 | 1220 | No other sets of primers were specific | | | |
| XCV2155-Rev1 | gcaacgccttggcgccgtaatgcagt | 65 |  |  |  |  |  |
| XCC2030-Fwd1 | TAAAGGTAAAGGTGGTGCCGGCCAACA | 65 | 926 | No other sets of primers were specific | | | |
| XCC2030-Rev1 | cggtcatcttcagccatgccagcca | 65 |  |  |  |  |  |
| XCC3594-Fwd1 | CGGCGCAATTACGTGACTGGGAACA | 65 | 633 | No other sets of primers were specific | | | |
| XCC3594-Rev1 | aacgcggcattgaacacgcattgctg | 65 |  |  |  |  |  |
| XCV2310-Fwd1 | ATTCCAATCTCGACCGAACGCTCGACG | 65 | 1203 | No other sets of primers were specific | | | |
| XCV2310-Rev1 | cctccatcccttggcctcaattcaagact | 65 |  |  |  |  |  |
| XCC0108-Fwd1 | CAACTCTCCTGCGTGATCTGCTCAAA | 58 | 399 | No other sets of primers were specific | | | |
| XCC0108-Rev1 | gccaccatcaggaagggacg | 58 |  |  |  |  |  |
| XAC2192-Fwd1 | AACGGCATATCGCGCTACAACGAGGC | 65 | 380 | No other sets of primers were specific | | | |
| XAC2192-Rev1 | tgttgaaccggttgaactcggccacc | 65 |  |  |  |  |  |
| XCC0397-Fwd1 | CGTCGATCTGCTGCAGATGCTCAAGGT | 65 | 1579 | No other sets of primers were specific | | | |
| XCC0397-Rev1 | aaatgttgttcgacgtttcaggcttgagatcggt | 65 |  |  |  |  |  |
| XCC1719-Fwd1 | CATCGTTTCGGTGCTGCCCGAGCAA | 65 | 1045 | No other sets of primers were specific | | | |
| XCC1719-Rev1 | ggtacgactcgttgcgatgcacggttt | 65 |  |  |  |  |  |
| XCC1340-Fwd1 | TCGGTCACGTTCAGCGCTGCGAGATT | 65 | 1438 | No other sets of primers were specific | | | |
| XCC1340-Rev1 | atcaacgcgatcaatgtcacccgcaatagc | 65 |  |  |  |  |  |
| XCC3595-Fwd1 | CTGCAGACCGGCGGCTACGTATTG | 65 | 800 | No other sets of primers were specific | | | |
| XCC3595-Rev1 | ggtgtaactcggcaggccgccaaaaaat | 65 |  |  |  |  |  |
| XCC3635-Fwd1 | ATACATGTTCTGCACCTCACCGGTCAGGTT | 65 | 1178 | No other sets of primers were specific | | | |
| XCC3635-Rev1 | agtgcgctcaagctgggcgccaaata | 65 |  |  |  |  |  |
| XCC4052-Fwd1 | TAATAGGCACGGCCGTTGTCGAGCTGC | 65 | 1307 | No other sets of primers were specific | | | |
| XCC4052-Rev1 | caagctggattacagccagaacgccg | 65 |  |  |  |  |  |
| XCC4237-Fwd1 | AAGGTCGGTAGCGGCTCGCAGGTATT | 65 | 1163 | No other sets of primers were specific | | | |
| XCC4237-Rev1 | cacccattcgccatccacgcggaa | 65 |  |  |  |  |  |
| XCC0304-Fwd1 | TCAGAAGTGGTAGGTCACCACCAGCC | 65 | 452 | No other sets of primers were specific | | | |
| XCC0304-Rev1 | cgcctggatgccaatgtcacctacaac | 65 |  |  |  |  |  |
| XCC3518-Fwd1 | CTCCCCTTCGTTGCCGAGTGTGTCCAT | 65 | 1300 | No other sets of primers were specific | | | |
| XCC3518-Rev1 | ggcccacatcgccattccagtcgaag | 65 |  |  |  |  |  |
| XCC4162-Fwd1 | CGCAGCAGCAGCGCCAGTTGGTA | 65 | 912 | No other sets of primers were specific | | | |
| XCC4162-Rev1 | acaccggcccgttgcgccatca | 65 |  |  |  |  |  |
| XCC0305-Fwd1 | ATGTAGGGCGCCAGCATGCTGGTGTA | 65 | 1211 | No other sets of primers were specific | | | |
| XCC0305-Rev1 | cggcatccccgatgaaggctcgtt | 65 |  |  |  |  |  |
| XCC2046-Fwd1 | ACTTCAACAACAAGCTGTACTACAACAGCTACGAA | 65 | 784 | No other sets of primers were specific | | | |
| XCC2046-Rev1 | atgcgatgcccacagcacccagtg | 65 |  |  |  |  |  |
| XCC0394-Fwd1 | AAGTTGCAACGTATTGGCAGTGTCCGCATA | 65 | 837 | No other sets of primers were specific | | | |
| XCC0394-Rev1 | cgtagatgccaaccgagcccacttc | 65 |  |  |  |  |  |
| XCC0120-Fwd1 | GACATCGGCAAGTTTCCCGATCTGAATCTG | 60 | 1414 | No other sets of primers were specific | | | |
| XCC0120-Rev1 | GTGTCGAAGGTGTTGCTCACCGACTG | 60 |  |  |  |  |  |
| XCC2867-Fwd1 | AACTGCGCAATGGTGTTCCGGTCTGCGTTT | 65 | 729 | No other sets of primers were specific | | | |
| XCC2867-Rev1 | gacgtccaggtgaagggctcgagtg | 65 |  |  |  |  |  |
| XAC3620-Fwd1 | ATCGTAGGGCTCGCGCGTGTCGGA | 65 | 830 | No other sets of primers were specific | | | |
| XAC3620-Rev1 | ctccatcgccaccaacctgctcgatt | 65 |  |  |  |  |  |
| XAC3201-Fwd1 | GCAACGGCTTGCCATCCACGTTGCAAT | 65 | 1886 | No other sets of primers were specific | | | |
| XAC3201-Rev1 | tttctgcttcccgatggtgggggac | 65 |  |  |  |  |  |
| XAC3077-Fwd1 | CGTATCGCAGGTACCGCCTATAGTGC | 65 | 1355 | No other sets of primers were specific | | | |
| XAC3077-Rev1 | gccataggccaccagggtattactgga | 65 |  |  |  |  |  |
| XAC2193-Fwd1 | GCCTGGCGGCGGTCTCCCAACAA | 65 | 790 | No other sets of primers were specific | | | |
| XAC2193-Rev1 | ggcgcggcgcaagggcgtataga | 65 |  |  |  |  |  |
| XAC0291-Fwd1 | AGCTTGCCATCGGCAATGTCGGGACG | 65 | 1231 | No other sets of primers were specific | | | |
| XAC0291-Rev1 | gaagacggctattacgtgcgcagatacatc | 65 |  |  |  |  |  |
| XAC4062-Fwd1 | CCGCCTGCGTTCGCTGGAGCAATTCA | 65 | 1442 | No other sets of primers were specific | | | |
| XAC4062-Rev1 | ccaaaatcctgccccgcattgacgatgaagatatt | 65 |  |  |  |  |  |
| XAC2185-Fwd1 | CAACGCAAGGCGCGACCAACGTGAAAGAA | 60 | 1417 | No other sets of primers were specific | | | |
| XAC2185-Rev1 | ATCGAAGATCGCCGCGCTGTAGCTCTG | 60 |  |  |  |  |  |
| XAC3498-Fwd1 | ATTCGCATCAGCGGTTCGAAGCCGATTGGAA | 65 | 1089 | No other sets of primers were specific | | | |
| XAC3498-Rev1 | ttgagctgcgccgaccatgcctggtt | 65 |  |  |  |  |  |
| XAC0852-Fwd1 | CAAGTTTCTGTTCGACGGTCTGCCGATCA | 65 | 1470 | No other sets of primers were specific | | | |
| XAC0852-Rev1 | aaagccgctgccatgcgcgtattgcaact | 65 |  |  |  |  |  |
| XAC3613-Fwd1 | TATGCCTTATCTGCTGTCTCTTGCCGTGTTGT | 65 | 1374 | No other sets of primers were specific | | | |
| XAC3613-Rev1 | tgtcgccgctggatcaagcaggttgtaact | 65 |  |  |  |  |  |
| XAC3050-Fwd1 | GCTTTTTTCGGAACCCCCGGCAGGCAAA | 65 | 831 | No other sets of primers were specific | | | |
| XAC3050-Rev1 | ggtgccattgcaaagcgcaaagcgac | 65 |  |  |  |  |  |
| XCV3187-Fwd1 | TCCAACCGCAGGCTCGCCGGCAATTA | 65 | 1285 | No other sets of primers were specific | | | |
| XCV3187-Rev1 | ggccgggctgatacaggtaagcc | 65 |  |  |  |  |  |
| XOO2829-Fwd1 | CGTCACCGTGGCAAGGTCCGCGAT | 65 | 824 | No other sets of primers were specific | | | |
| XOO2829-Rev1 | atcgatgacgtcggccggcaagcta | 65 |  |  |  |  |  |
| *pilL*-Fwd1 | CAGGTCATACACGTTCCGCATCAGATCCAACT | 65 | 1221 | No other sets of primers were specific | | | |
| *pilL*-Rev1 | CCGCTGGAACACATGCTGCGCAACTC | 65 |  |  |  |  |  |
| *pilS*-Rev1 | TGGTGCGCTGGCATTGCTGGTGGATGATCT | 65 | 739 | No other sets of primers were specific | | | |
| *pilS*-Fwd1 | ATCCTGCTGCGGCTGCGCTACGGCA | 65 |  |  |  |  |  |
| *pilU*-Rev1 | TCGAACAGGCTCTGGTCGAAGGTGCG | 65 | 977 | No other sets of primers were specific | | | |
| *pilU*-Fwd1 | ATGAGCACCATCGACTTCACCTCCTTCC | 65 |  |  |  |  |  |
| *pilA*-Rev1 | GAAGTTGGGTGTCGTCGATTTCGGAACTG | 65 | 360 | No other sets of primers were specific | | | |
| *pilA*-Fwd1 | GCGTGTTCGGCATCCTCGCTGCGAT | 65 |  |  |  |  |  |
| *xadA2*-Rev1 | ATGAAGCGAATTTATCTGGAGGCTGGTCGC | 65 | 1015 | No other sets of primers were specific | | | |
| *xadA2*-Fwd1 | TTCGGAAACACGTTGGAATCGTAGCCAATG | 65 |  |  |  |  |  |
| XCV2103-Fwd1 | TTCCGGGCAGCGTGGACATCAGCCAG | 65 | 1570 | No other sets of primers were specific | | | |
| XCV2103-Rev1 | CCGATGTCGGTGATGTTGAGCGTGCC | 65 |  |  |  |  |  |
| *fhaB-*Fwd1 | TGATCCTCAACAACAGTGCGCAGATCTCCAAG | 65 | 2596 | No other sets of primers were specific | | | |
| *fhaB*-Rev1 | ATCGTGCCGCTATCCAATGCAATCGCACCGGCG | 65 |  |  |  |  |  |
| *fhaB1*-Rev1 | CGCAGTGGTGAGGTTGGCAGCCTA | 65 | 1354 | No other sets of primers were specific | | | |
| *fhaB1*-Fwd1 | TGATCCTCAACAACAGTGCGCAGATCTCCAAG | 65 |  |  |  |  |  |
| *fhaB2*-Fwd1 | GTCTGCGACACGCGATACGCAAGTG | 65 | 1238 | No other sets of primers were specific | | | |
| *fhaB2*-Rev1 | ATCGTGCCGCTATCCAATGCAATCGCACCG | 65 |  |  |  |  |  |
| XAC1816-Rev1 | TTCAGCCCAGACTGCTGCAACTCGGT | 65 | 424 | No other sets of primers were specific | | | |
| XAC1816-Fwd1 | TACAGCCAGCAGAAGGTCAACAGCAGCTA | 65 |  |  |  |  |  |
